# Increased Proportion of CD226^+^ B Cells Is Associated With the Disease Activity and Prognosis of Systemic Lupus Erythematosus

**DOI:** 10.3389/fimmu.2021.713225

**Published:** 2021-07-21

**Authors:** Miki Nakano, Masahiro Ayano, Kazuo Kushimoto, Shotaro Kawano, Kazuhiko Higashioka, Shoichiro Inokuchi, Hiroki Mitoma, Yasutaka Kimoto, Mitsuteru Akahoshi, Nobuyuki Ono, Yojiro Arinobu, Koichi Akashi, Takahiko Horiuchi, Hiroaki Niiro

**Affiliations:** ^1^ Department of Medicine and Biosystemic Science, Kyushu University Graduate School of Medical Sciences, Fukuoka, Japan; ^2^ Department of Cancer Stem Cell Research, Kyushu University Graduate School of Medical Sciences, Fukuoka, Japan; ^3^ Department of Internal Medicine, Kyushu University Beppu Hospital, Beppu, Japan; ^4^ Department of Medical Education, Kyushu University Graduate School of Medical Sciences, Fukuoka, Japan

**Keywords:** systemic lupus erythematosus, CD226, B cells, lupus low disease activity, lupus nephritis

## Abstract

**Background:**

CD226, an activating receptor expressed on the surface of natural killer (NK) cells and T cells, is also seen on B cells and CD226 polymorphism is associated with systemic lupus erythematosus (SLE). Because the specific roles of CD226^+^ B cells in SLE are still unknown, we investigated the association of CD226^+^ B cells with SLE.

**Methods:**

We measured CD226 expression on B cells and its subsets using flow cytometry in 48 SLE patients and 24 healthy controls (HCs). We assessed the relationships between CD226^+^ B cells and SLE Disease Activity Index 2000 (SLEDAI-2K), clinical manifestations, laboratory data, and prognosis after 12 months.

**Results:**

The proportions of CD226^+^ cells in whole B cells and all its subsets were significantly higher in SLE patients than HCs. In SLE patients, the proportions of CD226^+^ B cells and CD226^+^ switched-memory (SM) B cells were significantly correlated with SLEDAI-2K scores and anti-dsDNA antibody titers, and negatively correlated with serum complement levels. Moreover, basal percentages of CD226^+^ B cells and CD226^+^ SM B cells were low in patients who were in Lupus Low Disease Activity State after 12 months. In patients with renal involvement, the proportion of CD226^+^ B cells increased. Additionally, the proportion of CD226^+^ B cells was higher in patients who were not in complete renal remission after 12 months.

**Conclusions:**

Increased proportion of CD226^+^ B cells was associated with disease activity and prognosis of SLE. CD226^+^ B cells may be a useful biomarker for the management of SLE.

## Introduction

Systemic lupus erythematosus (SLE) is a complex autoimmune disease with various clinical manifestations and its management is often difficult (1–3). Recently, the concept of treat-to-target (T2T) in SLE has been established, and it has been more important to monitor disease activity and predict the prognosis of SLE (3–5). Because of this, many studies have tried to find potential novel biomarkers from the molecules involved in the pathogenesis of SLE (6, 7).

Although the pathogenesis of SLE is not yet fully understood, it is known that genetic factors contribute to the development of SLE (2, 8–10) and that various immune cells are involved in the immune responses in SLE (11). In particular, B cells play an important role in the pathogenesis of SLE by autoantibody and cytokine production and antigen presentation (8, 11–13). Some B cell phenotypes have been reported as useful biomarkers for disease activity of SLE (7, 13); however, these have not yet been established.

Several genome-wide association studies have reported the importance of the CD226 gene in the susceptibility to SLE in multiple ancestries (14–17). CD226 is expressed on the cell surface of T cells, natural killer (NK) cells, B cells, monocytes, and platelets (18). It also acts as an activating receptor and mediates cytotoxicity and has been reported to modulate various immune functions as well (18–21). Moreover, several studies have shown the involvement of CD226 in autoimmune diseases such as SLE (22), rheumatoid arthritis (23), and systemic sclerosis (24). On the other hand, the immune functions of CD226 on B cells remain unclear, although a recent study suggested CD226 was involved in adaptive immune responses of B cells (25).

This study aimed to reveal the involvement of CD226 on B cells in the pathogenesis of SLE by measuring CD226 expression on B cells using flow cytometry in SLE patients and by assessing the relationship between CD226-expressing B cells and the disease activity, clinical manifestations, and prognosis of SLE.

## Materials and Methods

### Study Population

We investigated 48 Japanese patients who were treated for SLE at the Kyushu University hospital between 2018 and 2020. We included patients who fulfilled at least four of the American College of Rheumatology revised criteria for SLE (26) and had no other autoimmune diseases, infections, or cancers. Some patients were treated with corticosteroids, hydroxychloroquine, and immunosuppressive drugs, alone or in combination. Of these 48 SLE patients, we were able to assess 5 patients both before and after treatment. We also investigated 24 healthy controls (HCs).

This study was approved by the ethics committee of Kyushu University Hospital (approval number 2019-481) in accordance with the Helsinki Declaration. All participants gave written informed consent.

### Data Collection

We obtained the following information from the medical records of the patients: demographic data, clinical manifestations, medications, and laboratory findings including C3, C4, and anti-dsDNA antibody titers at baseline and after 12 months. The serum anti-dsDNA antibody titers were measured using chemiluminescence enzyme immunoassay, and the positive cut-off value used was >10 IU/ml. Disease activity was assessed by the SLE Disease Activity Index 2000 (SLEDAI-2K) (27) and the clinical SLEDAI-2K (28), with active SLE being defined as having a SLEDAI-2K score of ≥11 (29). The clinical SLEDAI-2K (28) is obtained by excluding the serological descriptors such as increased anti-dsDNA antibodies and low complement from the SLEDAI-2K. Clinical manifestations were classified based on the SLEDAI-2K descriptors (27), and renal manifestations were evaluated using the renal SLEDAI-2K scores (30), which were the renal descriptors of the SLEDAI-2K including urinary casts, hematuria, proteinuria, and pyuria. Active nephritis was defined as having a renal SLEDAI-2K score of ≥4. Previous lupus nephritis (LN) and neuropsychiatric systemic lupus erythematosus (NPSLE) were defined as having once before a renal SLEDAI-2K score of ≥4 and one or more neuropsychiatric descriptors of the SLEDAI-2K, respectively. Low disease activity (LDA) was assessed by the definitions of Lupus Low Disease Activity State (LLDAS): a SLEDAI-2K score of ≤4, with no activity in major organ systems (renal, central nervous system, cardiopulmonary, vasculitis, fever) and no hemolytic anemia or gastrointestinal activity; no new lupus disease activity compared with the previous assessment; physician global assessment (scale 0–3) of ≤1; a current prednisolone (or equivalent) dose of ≤7.5 mg/day; and well-tolerated standard maintenance doses of immunosuppressive drugs and approved biological agents (31). Complete renal remission (CR) was defined as proteinuria <500 mg/24 hours and serum creatinine levels within 10% from baseline (5).

### Flow Cytometric Analysis

Peripheral blood mononuclear cells (PBMCs) from SLE patients and HCs were isolated from the heparinized fresh blood by Lymphoprep (Stemcell Technologies, Vancouver, Canada). PBMCs obtained were pre-treated with FcR blocking reagent (Miltenyi Biotec, Bergisch Gladbach, Germany) and stained with the following fluorescence-conjugated anti-human antibodies: anti-CD3-PE-Cyanine (Cy) 7 (SK7), anti-CD4-Brilliant Violet (BV) 421 (RPA-T4), anti-CD8a-BV510 (RPA-T8), anti-CD19-APC (HIB19), anti-CD56-FITC (HCD56), anti-CD3-PerCP-Cy5.5 (UCHT1), anti-CD19-BV421 (HIB19), anti-CD20-PE-Cy7 (2H7), anti-CD27-BV510 (O323), anti-IgD-FITC (IA6-2), anti-CD38-APC-Cy7 (HIT2; all from BioLegend, San Diego, CA, USA), and anti-CD226-PE (DX11; Miltenyi Biotec). Isotype control antibodies (BD Biosciences, San Jose, CA, USA) were used to determine the level of background staining. Samples were analyzed with the FACS Aria II flow cytometer (BD Biosciences). Data analysis was performed using the FlowJo Software (Tree Star, Ashland, OR, USA). CD4^+^ T cells (CD3^+^CD4^+^), CD8^+^ T cells (CD3^+^CD8^+^), NK cells (CD3^−^CD56^+^), B cells (CD3^−^CD19^+^), naive B cells (CD3^−^CD19^+^IgD^+^CD27^−^), IgD^+^-memory B cells (CD3^−^CD19^+^IgD^+^CD27^+^), switched-memory (SM) B cells (CD3^−^CD19^+^IgD^−^CD27^+^), and plasmablasts (CD3^−^CD19^+^CD20^−^CD38^++^) were all assessed by flow cytometry.

### Statistical Analysis

Data are presented as the median and interquartile range unless otherwise stated. Differences between two groups were analyzed using the Student’s t-test for normally distributed continuous variables or using the Mann–Whitney *U* test for non-normally distributed variables. The relations between two continuous variables were analyzed using Spearman’s rank correlation. All tests were two-tailed and *P*-values < 0.05 were considered significant. All analyses were performed using the JMP software, version 15 (SAS Institute, Cary, NC, USA).

## Results

### The Proportions of CD226^+^ Cells in All B Cell Subsets Increased in SLE Patients

**Figure 1:**
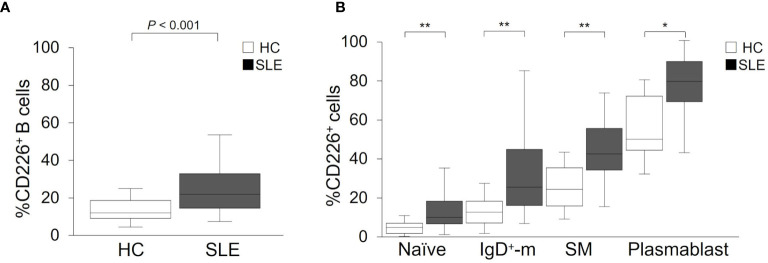
CD226 expression on B cell subsets in SLE patients and healthy controls (HCs). Proportions of CD226^+^ cells in B cells **(A)**, naive B cells, IgD^+^-memory (IgD^+^-m) B cells, switched-memory (SM) B cells, and plasmablasts **(B)** were compared between SLE patients and HCs. The median (interquartile range) proportions of CD226^+^ cells from SLE patients and HCs were 21.9% (14.4–32.9) and 11.9% (9.0–18.5) in B cells; 10.6% (7.4–18.7) and 5.4% (2.4–7.5) in naive B cells; 25.9% (16.6–44.9) and 13.2% (7.6–18.8) in IgD^+^-m B cells; 42.6% (34.5–55.5) and 24.8% (16.4–35.6) in SM B cells; 79.3% (69.0–89.3) and 50.0% (44.6–71.8) in plasmablasts. Box plots represent the median values, interquartile ranges, and the range of values. Statistical differences among groups were evaluated using the Mann–Whitney *U* test (*P < 0.005, **P < 0.001).

To investigate the involvement of CD226 in the pathogenesis of SLE, we first measured CD226 expression on PBMCs using flow cytometry in 48 SLE patients (mean age, 41.4 years; 43 females) and 24 HCs (mean age, 38.0 years; 23 females). No significant differences were found between SLE patients and HCs in terms of age and gender. The baseline characteristics of the SLE patients are described in **Table 1**. The proportions of CD226^+^ B cells and CD226^+^ CD8^+^ T cells were significantly higher in SLE patients than in HCs (**Figure 1A** and **Supplementary Figure 1A**), whereas those of CD226^+^ CD4^+^ T cells and CD226^+^ NK cells were almost the same between the two groups (**Supplementary Figures 1B, C**).

**Table 1:** Baseline characteristics of SLE patients.

Characteristics	
Age, mean (S.D.), years	41.4 (15.2)
Female, *n* (%)	43 (90)
Disease duration, median [IQR], years	11.0 [4–21]
Previous LN, *n* (%)	18 (38)
Previous NPSLE, *n* (%)	12 (25)
SLE disease activity	
SLEDAI-2K score, median [IQR]	3 [0.5–8.8]
SLEDAI-2K ≤ 3, *n* (%)	24 (50)
SLEDAI-2K 4–10, *n* (%)	14 (29)
SLEDAI-2K ≥ 11, *n* (%)	10 (21)
clinical SLEDAI-2K score, median [IQR]	2 [0–6]
Clinical manifestations, *n* (%)	
Renal	16 (33)
Mucocutaneous	14 (29)
Musculoskeletal	6 (13)
Hematological	4 (8)
Neuropsychiatric	3 (6)
Increased anti-dsDNA antibodies, *n* (%)	18 (38)
Anti-dsDNA antibody titer, median [IQR]	43.1 [19.7–174.5]
Low complement, *n* (%)	22 (46)
C3, median [IQR], mg/dl	78.5 [66–94.5]
C4, median [IQR], mg/dl	13.5 [10–19]
Corticosteroid use, *n* (%)	41 (85)
Prednisolone equivalent dose, median [IQR], mg/day	6.5 [5–12.5]
HCQ use, *n* (%)	9 (19)
Immunosuppressive agent use, *n* (%)	33 (69)

IQR, interquartile range; LN, lupus nephritis; NPSLE, neuropsychiatric systemic lupus erythematosus; SLEDAI-2K, systemic lupus erythematosus disease activity index in 2000; HCQ, hydroxychloroquine.

To reveal whether increased proportions of CD226^+^ cells occurred in a particular B cell subset, we assessed CD226 expression on each subset: naive B cells, IgD^+^-memory B cells, SM B cells, and plasmablasts. In both SLE patients and HCs, the proportions of CD226^+^ cells increased in differentiated B cells, such as SM B cells and plasmablasts (**Figure 1B**). Interestingly, the proportions of CD226^+^ cells in all B cell subsets were higher to same extent in SLE patients compared with HCs (**Figure 1B**). Thus, the proportions of CD226^+^ B cells increased in SLE patients and such increases were seen in all B cell subsets, with no specificity to a particular subset.

### CD226^+^ B Cells Are Associated With Disease Activity

**Figure 2:**
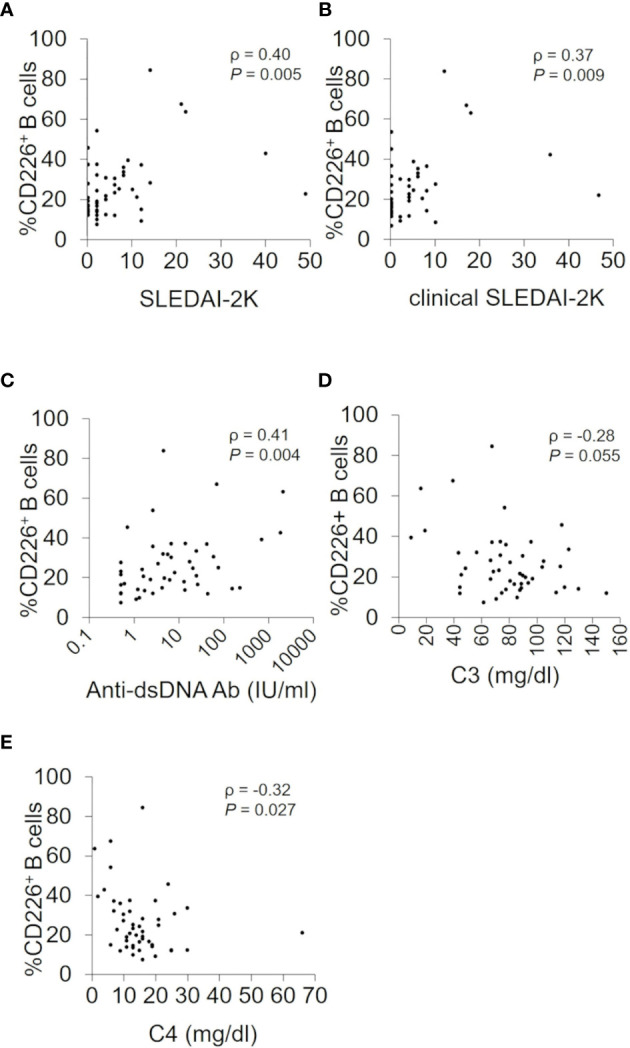
Associations between CD226^+^ B cells and SLEDAI-2K, clinical SLEDAI-2K, anti-dsDNA antibody titers, serum C3, and C4 levels. Correlations between the percentage of CD226^+^ B cells and SLEDAI-2K scores **(A)**, clinical SLEDAI-2K **(B)**, anti-dsDNA antibody (Ab) titers **(C)**, the serum C3 **(D)**, and C4 levels **(E)** in SLE patients. Each data point represents a single subject. Correlation analyses were evaluated using Spearman’s rank correlation. SLEDAI-2K, SLE Disease Activity Index 2000.

We studied the relation between CD226^+^ B cells and disease activity of SLE. We first confirmed that the proportion of CD226^+^ B cells had no obvious correlation with prednisolone equivalent dose (ρ = 0.09; *P* = 0.54) and were almost the same between SLE patients with immunosuppressive agents and those without immunosuppressive agents [23.0% (15.5–31.7) *vs* 21.4% (12.1–37.0); *P* = 0.76]. The percentage of CD226^+^ B cells had a significant correlation with SLEDAI-2K scores (ρ = 0.40; *P* = 0.005) and clinical SLEDAI-2K scores (ρ = 0.37; *P* = 0.009) (**Figures 2A, B**). This percentage also had a significant positive correlation with anti-dsDNA antibody titers (ρ = 0.41; *P* = 0.004) (**Figure 2C**) and an inverse correlation with serum levels of C3 (ρ = −0.28; *P* = 0.055) and C4 (ρ = −0.32; *P* = 0.027) (**Figures 2D, E**). Across all B cell subsets, the percentage of CD226^+^ SM B cells was also significantly correlated positively with SLEDAI-2K scores (ρ = 0.36; *P* = 0.012), clinical SLEDAI-2K scores (ρ = 0.30; *P* = 0.037), and anti-dsDNA antibody titers (ρ = 0.47; *P* = 0.001) and inversely correlated with C3 (ρ = −0.36; *P* = 0.011) and C4 levels (ρ = −0.39; *P* = 0.007). Similarly, the percentage of CD226^+^ plasmablasts was correlated positively but non-significantly with SLEDAI-2K scores (ρ = 0.27; *P* = 0.066), clinical SLEDAI-2K scores (ρ = 0.27; *P* = 0.059), and anti-dsDNA antibody titers (ρ = 0.27; *P* = 0.066), whereas CD226 expression on the other B cell subsets had no obvious relation to such parameters (**Supplementary Table 1**).

We also assessed the association between CD226^+^ B cells and clinical manifestations of SLE and found that the proportion of CD226^+^ B cells was significantly higher in patients with renal, musculoskeletal, and/or hematological manifestations (**Supplementary Table 2**). Thus, CD226^+^ B cells are associated with disease activity and clinical manifestations.

### CD226^+^ B Cells Are Decreased After Improvement of the Disease Activity With Treatment

**Figure 3:**
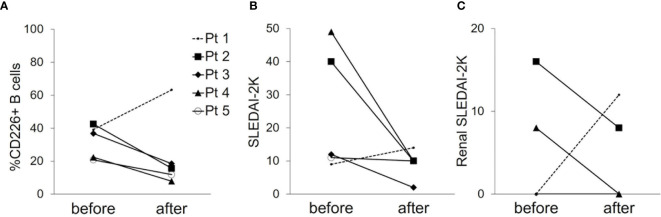
Changes in the frequency of CD226^+^ B cells and disease activity before and after treatment. The frequency of CD226^+^ B cells **(A)**, SLEDAI-2K scores **(B)**, and renal SLEDAI-2K scores **(C)** before and after treatment in 5 SLE patients are shown. Each data point represents a single subject. SLEDAI-2K, SLE Disease Activity Index 2000.

To further investigate the relation of CD226^+^ B cells and disease activity of SLE, we examined CD226^+^ B cells in 5 patients with active SLE before and after treatment (median follow-up duration, 1.0 month). In 4 SLE patients who had improved SLEDAI-2K scores and renal SLEDAI-2K scores after treatment, the frequency of CD226^+^ B cells decreased after medication (**Figures 3A–C**, shown in solid lines). In contrast, the frequency of CD226^+^ B cells increased in one SLE patient who had impaired disease activity (**Figures 3A–C**, shown in dashed lines). When we measured CD226 expression on each B cell subset in the same patients, the frequency of each CD226^+^ B cell subset showed no significant differences before and after treatment (data not shown). These findings suggest that CD226^+^ B cells reflect disease activity.

### CD226^+^ B Cells Can Predict Disease Prognosis After 12 Months

**Figure 4:**
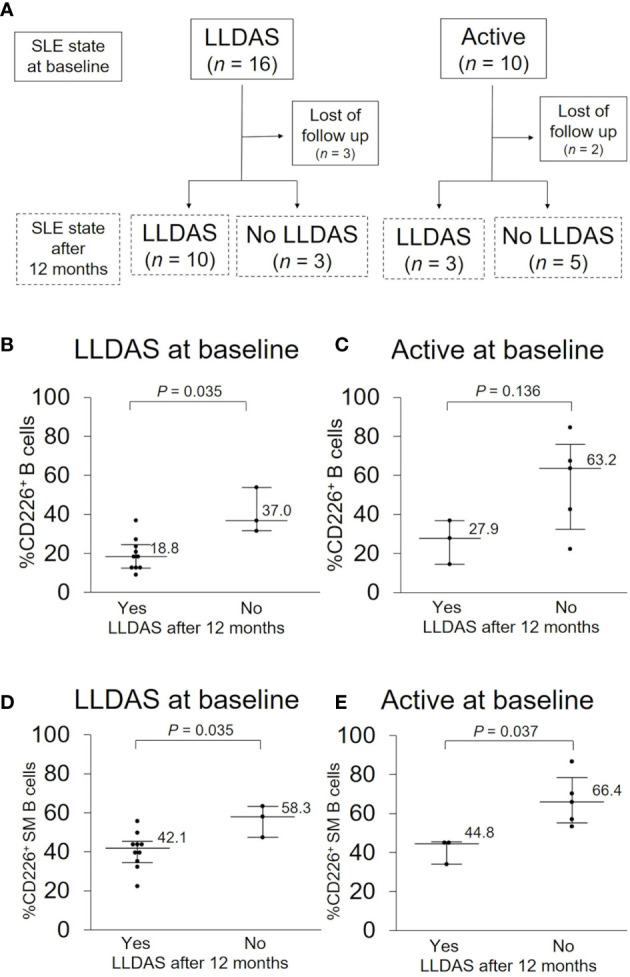
CD226^+^ B cells at baseline and disease prognosis after 12 months. **(A)** SLE activity state at baseline and after 12 months. In each activity state at baseline, basal proportions of CD226^+^ cells in B cells **(B, C)** and switched-memory (SM) B cells **(D, E)** were compared between patients who were in Lupus Low Disease Activity State (LLDAS) after 12 months and patients who were not. Each data point represents a single subject. Horizontal lines show the median and error bars represent interquartile ranges. Statistical differences among groups were evaluated using the Mann–Whitney *U* test.

Because SLE treatment should aim at remission or low disease activity and prevention of flares in all organs in 2019 EULAR recommendation (5), we assessed the total disease activity and prognosis first. Of the 48 SLE patients examined, 10 were in an active state and 16 were in LLDAS at baseline, and the percentage of CD226^+^ B cells was higher in an active state than in LLDAS [32.4% (19.3–64.2) *vs* 18.8% (13.4–30.7); *P* = 0.078]. To determine if CD226^+^ B cells at baseline can predict disease prognosis after 12 months, we compared the proportion of CD226^+^ B cells at baseline between patients who were in LLDAS after 12 months and those who were not (**Figure 4A**). In LLDAS patients at baseline, the proportion of CD226^+^ B cells and CD226^+^ SM B cells was significantly lower in patients who remained in LLDAS after 12 months than in patients who were not (**Figures 4B, D**). In active state patients at baseline, the percentage of CD226^+^ cells in B cells, especially SM B cells was low in patients who achieved LLDAS after 12 months (**Figures 4C, E**). These results suggest that CD226^+^ B cells, especially CD226^+^ SM B cells, are associated with SLE outcomes.

### CD226^+^ B Cells Can Predict Renal Prognosis After 12 Months

**Figure 5:**
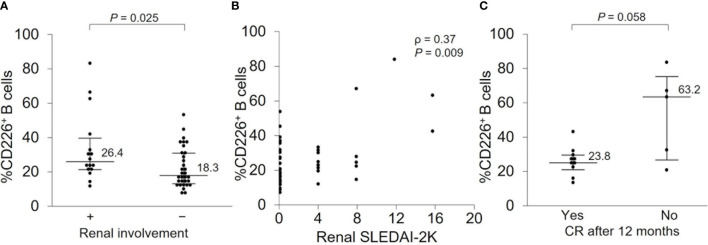
CD226^+^ B cells at baseline and renal prognosis after 12 months**. (A)** The frequency of CD226^+^ B cells was compared between SLE patients with renal involvement and those without. **(B)** Correlation between the percentage of CD226^+^ B cells and renal SLEDAI-2K scores. **(C)** In patients with renal involvement at baseline, the percentage of CD226^+^ B cells was compared between patients who achieved complete renal remission (CR) after 12 months and those who did not. Each data point represents a single subject. Horizontal lines show the median and error bars represent interquartile ranges. Statistical differences among groups were evaluated using the Mann–Whitney *U* test. Correlation analyses were evaluated using Spearman’s rank correlation. SLEDAI-2K, SLE Disease Activity Index 2000.

Because renal involvement is one of the major causes of morbidity and mortality in SLE patients (32) and our study included many patients with renal involvement, we wanted to further investigate the relationship between CD226^+^ B cells and renal manifestation. There were 16 patients with active nephritis at baseline, and they had a significantly elevated percentage of CD226^+^ B cells (**Figure 5A** and **Supplementary Table 2**) which had a significant correlation with renal SLEDAI-2K scores (ρ = 0.37; *P* = 0.009) (**Figure 5B**). To investigate the association of CD226^+^ B cells and renal prognosis, we then compared the percentage of CD226^+^ B cells between patients who achieved CR after 12 months and patients who did not. In patients who achieved CR after 12 months, the frequency of CD226^+^ B cells at baseline was lower than in patients who did not (**Figure 5C**). The percentages of CD226^+^ cells in each B cell subset were almost the same between the two groups (data not shown). These findings indicate that CD226^+^ B cells may be associated with renal involvement and prognosis.

## Discussion

In this study, we showed that the proportion of CD226^+^ B cells was significantly higher in SLE patients and was associated with disease activity and renal involvement. We also demonstrated that the proportion of CD226^+^ B cells predicted disease outcome and renal prognosis after 12 months.

CD226 is a costimulatory adhesion molecule expressed on NK cells and T cells which mediates cytotoxic signals (18–21). T cell immunoreceptor with immunoglobulin and immunoreceptor tyrosine-based inhibitory motif domain (TIGIT), a paired receptor of CD226, is a coinhibitory receptor that inhibits the activation of T cells and NK cells (33, 34). Both CD226 and TIGIT bind to CD112 and CD155, and TIGIT inhibits the interaction between CD155 and CD226 (33). Some studies have shown the involvement of CD226 and TIGIT in SLE (14–17, 22, 35). In mouse SLE models, treatment with the TIGIT-Ig fusion protein was effective for the reduction of autoantibody production and increase in survival rate (35). Regarding CD226, several studies have reported that the nonsynonymous rs763361 polymorphism in CD226 was associated with SLE in multiple ancestries (14–17). Furthermore, another study has shown that the proportion of CD226^+^ NK cells was decreased in active SLE patients, and this increased in some patients during therapy (22). In this previous study, CD226 expression was assessed in SLE patients with severe disease activity for a short period of time. CD226 is also expressed on the cell surface of B cells (25) which play a pivotal role in SLE (8, 11–13); however, little is known regarding the associations of CD226 on B cells with SLE. Therefore, our study focused on CD226 expression on B cells in SLE patients with various disease activity for a longer period of time.

In our study, the proportion of CD226^+^ B cells was significantly higher in SLE patients compared to HCs. Additionally, the percentage of CD226^+^ B cells was associated with disease activity and positively correlated with anti-dsDNA antibody titers. Across all B cell subsets, CD226^+^ SM B cells and CD226^+^ plasmablasts were associated with disease activity of SLE, consistent with the reports that showed SM B cells and plasmablasts increased in SLE patients and were associated with disease activity (36–39). Although we did not investigate the immune functions of CD226 on B cells in SLE, a recent study with healthy subjects reported that CD226^+^ B cells were upregulated by stimulation *via* toll-like receptor (TLR) 9 and were also involved in IL-10 and antibody productions (25). Furthermore, many studies have shown that both TLR9 and IL-10 play pivotal roles in B cell immunity in SLE (40–46). These findings suggest that CD226^+^ B cells, especially CD226^+^ SM B cells and CD226^+^ plasmablasts are involved in the pathogenesis of SLE.

Our analysis showed that the proportions of CD226^+^ cells increased in differentiated B cell subsets such as SM B cells and plasmablasts in SLE patients, consistent with a recent report involving HCs (25). B cells have been classified into several phenotypes based on differentiation stage (47), and their functions in immune responses are different (48–50). Other reports claim that the proportions of some B cell subsets such as SM B cells and plasmablasts are altered in SLE patients (13, 36–39, 51). In our study, however, the proportions of CD226^+^ cells in all B cell subsets were higher in SLE patients than in HCs, indicating that CD226 upregulation may not reflect alterations in the proportions of B cell subsets in SLE. A recent study reported that CD226^+^ B cells were upregulated by stimulation *via* TLR 9 (25). Given that TLR 9 expression has been known to increase in differentiated B cell subsets such as SM B cells and plasmablasts (43, 44), TLR 9 may affect the CD226 upregulation according to differentiation stage of B cells. Further analyses of the mechanism of CD226 upregulation on B cells are required to clarify this.

In this study, increased proportion of CD226^+^ B cells was associated with disease activity. Moreover, CD226^+^ B cells at baseline were low in patients who were in LLDAS after 12 months. According to the 2019 EULAR recommendation, SLE treatment should aim for remission or LDA monitoring disease activity (5). Among various definitions of LDA, LLDAS has been used in many studies (31, 52, 53). While there are many biomarkers for monitoring disease activity (6, 7), there are no useful markers for predicting LLDAS. In our study, conventional biomarkers such as anti-dsDNA titers and complement levels were associated with disease activity; however, these biomarkers could not predict the prognosis after 12 months (data not shown). Our study showed that CD226^+^ B cells were associated with disease activity and the prognosis of SLE. Although some studies have tried to find B cell phenotypes associated with disease activity of SLE, these studies need to measure the expression of various surface molecules, but these were not established (13, 38, 39). Although our results are needed to be validated in a prospective study with a larger sample size, CD226^+^ B cells may be a candidate of a useful biomarker for disease activity and prognosis of SLE.

In this study, we further investigated the relationship between CD226^+^ B cells and renal manifestation. The results showed that the proportion of CD226^+^ B cells was higher in patients with renal involvement; this reflected renal disease activity. Among various clinical manifestations of SLE, LN is a major cause of morbidity and mortality (32). Therefore, it is recommended that treatment for LN should aim for CR (5, 54, 55). However, no useful biomarkers for predicting CR have yet been developed. In our study, we showed that patients with a higher percentage of CD226^+^ B cells at baseline did not achieve CR after 12 months. Although it is necessary to investigate the infiltration of CD226^+^ cells into the kidney and urinary analysis, these findings indicate the utility of CD226^+^ B cells as a biomarker for predicting renal outcome.

Our study had some limitations. First, this study was a single-center study with small sample size. Because our institution is a tertiary referral hospital, a relatively large number of patients with refractory manifestations, such as LN and NPSLE, were included in this study. Moreover, although we targeted patients with various disease activity and clinical manifestations, there were few active patients without renal manifestations in our study. There is a need to replicate this study with a larger sample size in a multicenter setting and assess the prognosis in active patients with other than renal manifestations. Second, the functions of CD226 on B cells in SLE are still unknown; further studies are required to reveal the mechanisms of CD226 on B cells in SLE. Lastly, this study was a retrospective study, and anti-dsDNA antibody titers using Farr assay, which is defined in SLEDAI-2K (27), were not measured. To confirm the association of CD226 on B cells with the prognosis of SLE, a prospective study needs to be performed as well.

In conclusion, we demonstrated that the proportion of CD226^+^ B cells increased in SLE patients and could be associated with disease activity and prognosis. These findings enable more precise control for the T2T strategy in SLE.

## Data Availability Statement

The original contributions presented in the study are included in the article/**Supplementary Material**.

## Ethics Statement

The studies involving human participants were reviewed and approved by the ethics committee of Kyushu University Hospital (approval number 2019-481). The patients/participants provided their written informed consent to participate in this study.

## Author Contributions

MN and MAy participated in study conception and design. MN, MAy, KK, SK, KH, and SI participated in data acquisition and analysis. MN, MAy, KK, HM, YK, MAk, NO, YA, KA, TH, and HN contributed to the interpretation of results. MN was a major contributor in writing the manuscript. All authors contributed to the article and approved the submitted version.

## Funding

This work was supported by Japan Society for the Promotion of Science [grant number JSPS KAKENHI 19K17887].

## Conflict of Interest

The authors declare that the research was conducted in the absence of any commercial or financial relationships that could be construed as a potential conflict of interest.
