# Supplementary material for: Increased Proportion of CD226+ B Cells Is Associated With the Disease Activity and Prognosis of Systemic Lupus Erythematosus
Source: Front Immunol. 2021 Jul 21;12:713225. doi: 10.3389/fimmu.2021.713225 (PMC8334729; doi:10.3389/fimmu.2021.713225)
Supplement: Supplementary file 1 [file DataSheet_1.docx]

Supplementary Material

# Supplementary Figures and Tables


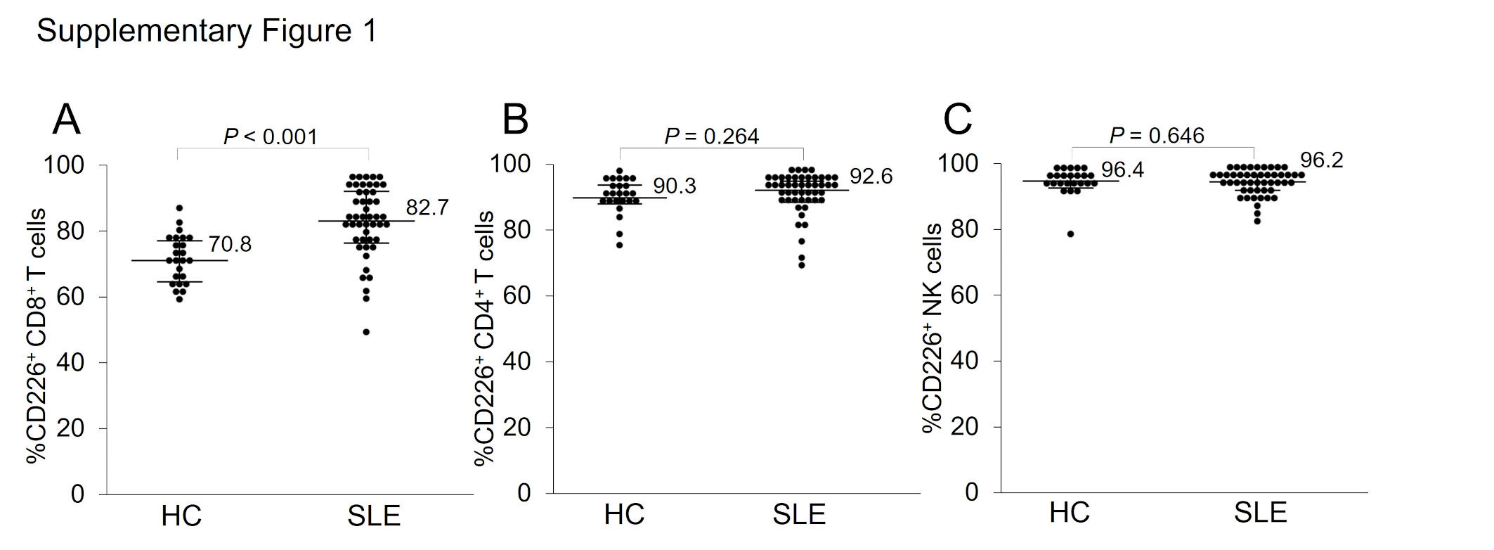


**Supplementary Figure 1.** CD226 expression on T cells and NK cells in SLE patients and healthy controls (HCs)

Proportions of CD226-expressing cells in CD8^+^ T cells (A), CD4^+^ T cells (B), and natural killer (NK) cells (C) were compared between SLE patients and HCs. Each data point represents a single subject. Horizontal lines show the median and error bars represent interquartile ranges. Statistical differences among groups were evaluated using the Mann–Whitney *U* test.

**Supplementary Table 1.** Associations between CD226 expression on each B cell subset and SLEDAI-2K and clinical SLEDAI-2K.

SLEDAI-2K clinical SLEDAI-2K

ρ, *P*-value ρ, *P*-value

CD226^+^ naive B cells 0.13, 0.38 0.10, 0.48

CD226^+^ IgD^+^-memory B cells 0.24, 0.11 0.20, 0.17

CD226^+^ SM B cells 0.36, 0.012 0.30, 0.037

CD226^+^ plasmablasts 0.27, 0.066 0.27, 0.059

Correlations between the percentage of CD226-expressing cells in each B cell subset and SLEDAI-2K scores and clinical SLEDAI-2K scores in SLE patients. Correlation analyses were evaluated using Spearman’s rank correlation. SLEDAI-2K: SLE Disease Activity Index 2000; SM: switched-memory.

**Supplementary Table 2.** CD226 expression on B cells in SLE patients with each clinical manifestation

Clinical manifestations median CD226^+^ B cells [IQR], % *P*-value

*n* (%) presence absence

Renal 16 (33) 26.4 [21.7–40.2] 18.3 [13.4–31.5] 0.025

Mucocutaneous 14 (29) 29.2 [11.8–38.2] 20.1 [14.6–30.5] 0.461

Musculoskeletal 6 (13) 36.2 [25.4–48.6] 20.1 [13.8–30.7] 0.024

Hematological 4 (8) 36.2 [25.1–60.0] 20.7 [14.0–31.3] 0.050

Neuropsychiatric 3 (6) 22.4 [8.9–42.5] 21.4 [14.5–32.6] 0.898

Proportions of CD226-expressing cells in B cells were compared between SLE patients with each clinical manifestation and those without. Statistical differences among groups were evaluated using the Mann–Whitney *U* test. IQR: interquartile range.
